# Dysbalance of Astrocyte Calcium under Hyperammonemic Conditions

**DOI:** 10.1371/journal.pone.0105832

**Published:** 2014-08-25

**Authors:** Nicole Haack, Pavel Dublin, Christine R. Rose

**Affiliations:** Institute of Neurobiology, Faculty of Mathematics and Natural Sciences, Heinrich Heine University, Duesseldorf, Germany; Albany Medical College, United States of America

## Abstract

Increased brain ammonium (NH_4_^+^/NH_3_) plays a central role in the manifestation of hepatic encephalopathy (HE), a complex syndrome associated with neurological and psychiatric alterations, which is primarily a disorder of astrocytes. Here, we analysed the influence of NH_4_^+^/NH_3_ on the calcium concentration of astrocytes *in situ* and studied the underlying mechanisms of NH_4_^+^/NH_3_-evoked calcium changes, employing fluorescence imaging with Fura-2 in acute tissue slices derived from different regions of the mouse brain. In the hippocampal *stratum radiatum*, perfusion with 5 mM NH_4_^+^/NH_3_ for 30 minutes caused a transient calcium increase in about 40% of astrocytes lasting about 10 minutes. Furthermore, the vast majority of astrocytes (∼90%) experienced a persistent calcium increase by ∼50 nM. This persistent increase was already evoked at concentrations of 1–2 mM NH_4_^+^/NH_3_, developed within 10–20 minutes and was maintained as long as the NH_4_^+^/NH_3_ was present. Qualitatively similar changes were observed in astrocytes of different neocortical regions as well as in cerebellar Bergmann glia. Inhibition of glutamine synthetase resulted in significantly larger calcium increases in response to NH_4_^+^/NH_3_, indicating that glutamine accumulation was not a primary cause. Calcium increases were not mimicked by changes in intracellular pH. Pharmacological inhibition of voltage-gated sodium channels, sodium-potassium-chloride-cotransporters (NKCC), the reverse mode of sodium/calcium exchange (NCX), AMPA- or mGluR5-receptors did not dampen NH_4_^+^/NH_3_-induced calcium increases. They were, however, significantly reduced by inhibition of NMDA receptors and depletion of intracellular calcium stores. Taken together, our measurements show that sustained exposure to NH_4_^+^/NH_3_ causes a sustained increase in intracellular calcium in astrocytes *in situ*, which is partly dependent on NMDA receptor activation and on release of calcium from intracellular stores. Our study furthermore suggests that dysbalance of astrocyte calcium homeostasis under hyperammonemic conditions is a widespread phenomenon, which might contribute to the disturbance of neurotransmission during HE.

## Introduction

Hepatic encephalopathy (HE) is a complex disorder resulting from liver failure, which is associated with neurological and psychiatric alterations and symptoms ranging from disturbance in fine motor coordination or intellectual function to unconsciousness and coma [1]. Strong evidence suggests that an increase in brain ammonium (NH_4_^+^/NH_3_) plays a critical role in in the manifestation of HE [2], [3]. Moreover, it is widely accepted that HE is primarily a disorder of astrocytes [2], [4]. Thus, NH_4_^+^/NH_3_-induced alterations in the function and properties of astrocytes are thought to represent the primary cause of HE.

Astrocytes are the only brain cells able to efficiently metabolize and detoxify NH_4_^+^/NH_3_ through the action of glutamine synthase, an enzyme almost exclusively located in astrocytes [5]. This reaction is central for the glutamate-glutamine cycle and glutamatergic as well as GABAergic neurotransmission in the healthy brain, and there is ample evidence that hyperammonemia not only affects both GABAergic and glutamatergic pathways [6]–[9], but also other neurotransmitter systems [10]. For example, altered glutamate/glutamine ratios and glutamate concentrations are consistently observed in patients and animal models of HE suggesting a disturbance of the glutamate-glutamine cycle [11]–[18]. Moreover, astrocyte swelling can be seen following exposure to NH_4_^+^/NH_3_ and this is considered to be the main cause of fatal cerebral oedema in acute HE [1], [19]–[24]. Astrocyte swelling leads to the production of reactive oxygen and nitrogen compounds, which in turn intensify the swelling [25].

Alterations in intracellular ion concentrations may also contribute to the observed cellular changes in HE. Perfusion of astrocytes with NH_4_^+^/NH_3_ causes immediate changes in their pH, consisting of an intracellular alkalinisation followed by pronounced intracellular acidification which develops in the continued presence of NH_4_^+^/NH_3_ [26]–[28]. A substantial increase in intracellular sodium concentration due to activation of sodium-potassium-chloride cotransport (NKCC1) was described in astrocytes upon application of NH_4_^+^/NH_3_ [28], [29]. Furthermore, astrocytes depolarize in response to alterations in extracellular potassium under hyperammonemic conditions [30], [31].

Several studies have found evidence that NH_4_^+^/NH_3_ application also affects glial calcium homeostasis, albeit with different characteristics depending on the model system used. Whereas a sustained calcium increase was reported from cultured cerebral rat astrocytes in response to acute perfusion with 5 mM NH_4_^+^/NH_3_ [32], this only caused a short and transient increase in the calcium concentration in astrocytes cultured from mouse cerebral cortex [33]. A recent study performed *in vivo*, reported increased and desynchronized calcium signaling in cortical astrocytes in response to injection of ammonia [31]. NH_4_^+^/NH_3_-induced calcium signals seem to represent a critical step in NH_4_^+^/NH_3_-induced osmotic and oxidative/nitrosative stress [25], [34] and can induce release of glutamate from cultured astrocytes, a mechanism which might contribute to increased extracellular levels of glutamate in brain tissue during HE [33], [35]–[37]. Calcium signaling in astrocytes mediates neuron-glia interaction and neuronal plasticity and its disturbance under hyperammonemic conditions may consequently contribute to altered neurotransmission in HE [38]–[40].

In the present study, we examined the effects of NH_4_^+^/NH_3_ on astrocyte calcium in acutely isolated tissue slices of the mouse brain, which represent a well-established model system for the study of the acute effects of NH_3_/NH_4_^+^ on brain cells [2]. Calcium was monitored performing ratiometric wide-field imaging with the fluorescent dye Fura-2. Our study shows that hyperammonemic conditions result in calcium increases in astrocytes in different regions of the brain and suggests NMDA receptor activation and release of calcium from intracellular stores as major mechanisms contributing to their generation. Because astrocyte calcium is an important mediator of neuron-glia interaction and neural plasticity, this dysbalance might contribute to the disturbance of neurotransmission during HE.

## Materials and Methods

### Ethics Statement

This study was carried out in strict accordance with the institutional guidelines of the Heinrich Heine University Duesseldorf, Germany, as well as the European Community Council Directive (86/609/EEC). All experiments were communicated to and approved by the Animal Welfare Office at the Animal Care and Use Facility of the Heinrich Heine University Duesseldorf, Germany (institutional act number: O52/05). In accordance with the German Animal Welfare Act (Tierschutzgesetz, Articles 4 and 7), no formal additional approval for the post mortem removal of brain tissue was necessary. For generation of acute slices, mice were anesthetized with CO_2_ and quickly decapitated (following the recommendation of the European Commission published in: Euthanasia of experimental animals, Luxembourg: Office for Official Publications of the European Communities, 1997; ISBN 92-827-9694-9).

### Preparation of Hippocampal Tissue Slices and Salines

Acute brain tissue slices were prepared from Balb/c mice (*mus musculus*) of both genders at postnatal days 14–16 (P14-16) using standard procedures. After decapitation of the animals, brains were quickly excised and transferred to ice-cold artificial cerebrospinal fluid (ACSF) composed of (in mM): 130 NaCl, 2.5 KCl, 2 CaCl2, 1 MgCl2, 1.25 NaH2PO4, 26 NaHCO3, and 20 glucose, bubbled with 95% O2 and 5% CO2, adjusted to a pH of 7.4. Parasaggital tissue slices (250 µm) of the hippocampus and cortex, and (30 µm) slices of the cerebellum were prepared using a vibratome (Microm HM650V, Thermo Fischer Scientific, Walldorf, Germany). Slices were transferred to ACSF at 34°C for 30 minutes. For selective vital staining of astrocytes [41], sulforhodamine 101 (SR101, 0.5–1 *µ*M) was added for 20 minutes to hippocampal and cortical slices during this period. We have shown in an earlier study, that with this procedure, SR101 selectively stains astrocytes in hippocampal slices preparations [42]. Slices were then maintained in ACSF at room temperature (RT, 19–22°C) until they were used for experiments, which were also performed at RT. While it has been reported that acute addition of SR101 for 10 minutes can induce periods of increased electrical activity in hippocampal slices [43], imaging measurements in astrocytes were only started at least 60 minutes after exposure to SR101.

Ammonia exists in two forms at physiological pH values, the uncharged form (NH_3_) and the ammonium ion (NH_4_^+^); in the following we will use the term NH_4_^+^/NH_3_ to reflect this situation. The pK_a_ of ammonia is 9.15 at 37°C and under physiological conditions, more than 98% of ammonia is present as NH_4_^+^. NH_4_^+^/NH_3_-containing salines were prepared by equimolar substitution of Na^+^. Nominally calcium-free saline was prepared by replacement of CaCl_2_ by MgCl_2_ and addition of 1 mM EGTA (ethylene glycol tetraacetic acid). For block of glutamine synthethase, tissue slices were pre-incubated with 100 µM MSO (methionine-S-sulfoximine) for three hours before commencing the imaging experiments.

All chemicals were purchased from Sigma-Aldrich (Munich, Germany), except for tetrodotoxin (Alomone Labs, Jerusalem, Israel or Biotrend Chemicals, Cologne, Germany).

### Imaging

Dynamic fluorescence imaging was performed using a widefield imaging system (TILL Photonics, Martinsried, Germany) attached to an upright microscope (Axioskop, Zeiss, Oberkochen, Germany) equipped with a 40x water-immersion-objective (PlanFI/IR, Olympus) and coupled to a cooled CCD camera (SensiCam QE, PCO, Kehlheim, Germany). For astrocyte identification, SR101 was excited at 575 nm, emission was collected above 590 nm. Cells were bulk-loaded by injection of the AM- (acetoxymethylester-) form of the calcium indicator dye Fura-2 or the pH indicator BCECF (250 µM; Teflabs; Invitrogen, Karlsruhe, Germany) into the brain slice preparations as described earlier [42], [44].

For ratiometric measurement of calcium, Fura-2 was alternately excited at 357 nm (isosbestic point) and 380 nm (calcium-sensitive wavelength) at 0.1 Hz. Emission (>440 nm) was collected in defined regions of interest (ROI) representing cell bodies of SR101-positive astrocytes or of Bergman glia cells. For pH_i_ measurements, BCECF emission intensities were collected between 490–575 nm following excitation at 452 nm and 488 nm. After standard dynamic background correction, the ratio of fluorescence emission (F_357_/F_380_ or F_488_/F_452_) was calculated for the individual ROIs and analyzed off-line using Microsoft Excel 2010 (Microsoft Corporation, Redmond, U.S.A) and OriginPro 8 G Software (OriginLab Corporation, Northhampton, MA, U. S. A.). Changes in BCECF ratio were transformed into pH according to calibrations described earlier [28].

Changes in the fluorescence ratio of Fura-2 were expressed as changes in calcium concentration based on an *in situ* calibration approach as reported before [45], [46]. To this end, slices were first bolus-loaded with Fura-2-AM in standard ACSF. Intracellular Fura-2 emission (F_357_ and F_380_ nm) was then recorded from defined regions of interest (ROIs) representing cell bodies of astrocytes, and the ratio (F_357_/F_380_ nm) was calculated. Subsequently, slices were perfused with a nominally calcium-free calibration saline containing in mM: 130 NaCl, 2.5 KCl, 3 MgCl_2_, 1.25 NaH_2_PO_4_, 26 NaHCO_3_, 20 glucose; bubbled with 95% O_2_, 5% CO_2_, resulting in a pH of 7.4. Furthermore, 5 µM of the calcium ionophore ionomycin, 5 µM monensin, 1 mM ouabain, 10 µM cyclopiazonic acid and 1 mM EGTA were added. Fura-2 emission was again recorded from astrocyte cell bodies after excitation at F_357_ and F_380_ nm, and the ratio was calculated, representing R_min_ (ratio at zero calcium). Subsequently, slices were perfused with calibration saline containing a calcium concentration of 10 mM (and no EGTA) to obtain fluorescence values and R_max_ at saturated Fura-2. These calibrations revealed an average R_min_ of 0.71±0.01 (*n* = 21 cells; *N* = 3 slices) and an R_max_ of 2.94±0.16 (*n* = 18 cells; *N* = 6 slices). Maximum emission at F_380_ in nominally calcium-free saline was 278±26 (arbitrary units), minimum emission at F_380_ under saturating conditions was 105±20. These values, together with a K_d_ of 224 nm [46] were then used to calculate calcium concentrations according to the equations provided before [45], [46].

A cell was considered to be responsive to NH_3_/NH_4_^+^ application, if its calcium concentration exceeded the average noise level of the baseline calcium concentration under control conditions by more than 2 standard deviations within 10 minutes after starting the NH_3_/NH_4_^+^ perfusion.

### Data presentation and statistics

Unless otherwise specified, data are expressed as means ± S.E.M and were statistically analyzed by one-tailed Student's t-test; Šidák correction was employed for multiple comparisons. p represents error probability, *n*.s. =  not significant, *p<0.05, **p<0.01, ***p<0.001. If not stated otherwise, *n* represents the number of cells and *N* the number of slices analyzed. Individual data points obtained from experiments and utilized for statistical analysis are supplied as Data S1.

## Results

### NH_4_^+^-induced changes in intracellular calcium in hippocampal astrocytes

**Figure 1:**
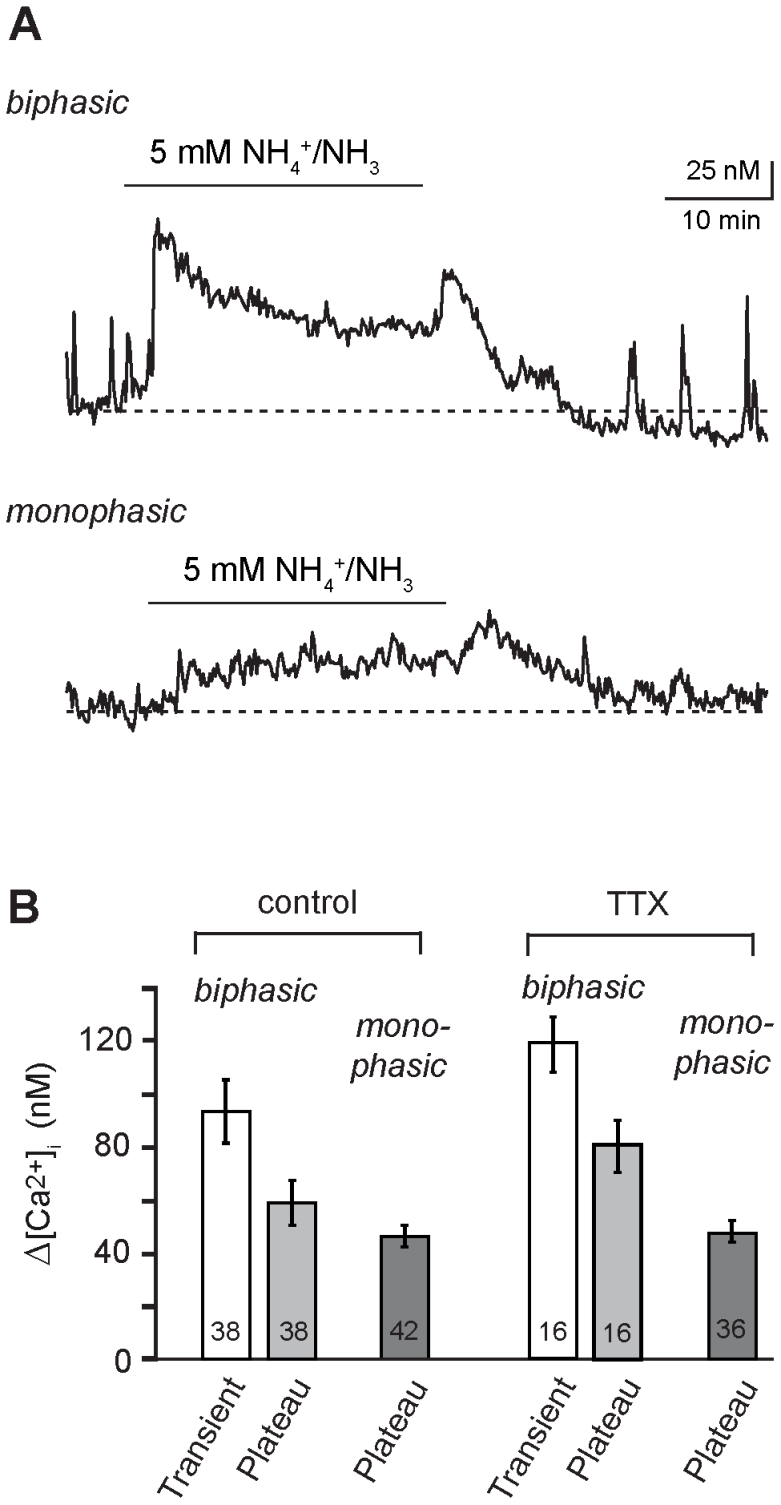
NH_4_^+^-induced changes in intracellular calcium in hippocampal astrocytes. **(A)** Calcium changes evoked by bath perfusion with 5 mM NH_4_^+^/NH_3_ for 30 minutes (indicated by bar) in two different SR101-positive astrocytes of the hippocampus. Upper trace: biphasic response consisting of a transient elevation followed by a plateau phase. Lower trace: monophasic response with a plateau phase only. Note the additional transient increase in calcium upon washout of NH_4_^+^/NH_3_ in both cells. **(B)** Histogram showing the mean peak amplitude ± S. E. M. of NH_4_^+^/NH_3_-induced transient and persistent (“plateau”) calcium changes in biphasic and monophasic cells in the absence (“control”) and presence of 0.5 µM tetrodotoxin (“TTX”). The number of cells is given within the bars. Response amplitudes in TTX are not different to those in the control.

Ratiometric imaging using the calcium-sensitive dye Fura-2 [44] revealed a baseline calcium concentration of 156.6±5.8 nM (*n* = 226, *N* = 32) in SR101-positive astrocytes of the CA1 area of the *stratum radiatum*. To study the influence of acute hyperammonemic conditions on the intracellular calcium concentration of hippocampal astrocytes, tissue slices were perfused with a saline containing 5 mM NH_4_Cl for periods of 30 minutes. Bath application of NH_4_^+^/NH_3_ induced a rise in the calcium concentration in the majority of astrocytes (88%) investigated (*n* = 91, *N* = 12). Generally, two types of responses could be distinguished in the astrocyte population, termed “biphasic” and “monophasic” response (Fig. 1). The response of a given astrocyte did neither correlate with its baseline calcium concentration, the pattern of spontaneous activity, nor the location of the cell in the *stratum radiatum* (distance from CA1 pyramidal cell layer or depth in the tissue slice).

In about half the responsive cells (48%), a biphasic calcium signal was observed upon NH_4_^+^/NH_3_ perfusion (“biphasic” response). It consisted of an initial transient increase in calcium concentration by 93.0±11.7 nM, which peaked within about 4 minutes upon NH_4_^+^/NH_3_ perfusion (Fig. 1A, upper trace). Calcium levels then slowly declined within 10–15 minutes to reach a stable plateau, which was maintained as long as the NH_4_^+^/NH_3_ was present. In the second group of astrocytes (52%), the initial transient response was missing and NH_4_^+^/NH_3_ perfusion induced a slow rise in the intracellular calcium concentration to a plateau level only (“monophasic” response; Fig. 1A, lower trace). The amplitude of the plateau, as determined at 20–30 minutes of NH_4_^+^/NH_3_ application, did not significantly differ between both types of cells (biphasic cells: 58.6±8.2 nM; monophasic cells: 46.0±3.8 nM; Fig. 1B). Pooling the data obtained in both cell types yielded an average of 52.0±4.4 nM for the amplitude of the persistent calcium increase in hippocampal astrocytes (*n* = 80, *N* = 12).

In both groups (biphasic as well as monophasic cells), removal of NH_4_^+^/NH_3_ was followed by another transient increase in calcium by 46.1±5.6 nM which lasted 4–6 minutes, and after which calcium recovered towards the baseline (Fig. 1A, B). Full recovery, however, was only observed in part of the cells (24%; Fig. 1A). Thus, as compared to the baseline before NH_4_^+^/NH_3_ application, calcium was increased by 40.6±4.3 nM (*n* = 80, *N* = 12) in the majority of astrocytes (75%) for at least 30 more minutes after removal of NH_4_^+^/NH_3_.

To study the influence of neuronal activity, we applied tetrodotoxin (TTX, 0.5 µM), a blocker of voltage-gated sodium channels. In the presence of TTX, the amplitude of NH_4_^+^/NH_3_-evoked calcium increases was not significantly different from the control without TTX both in biphasic (transient increase: 118.9±10.3 nM; plateau: 80.8±9.6 nM; *n* = 16, *N* = 8; Fig. 1B) and in monophasic cells (plateau: 47.4±3.9 nM; *n* = 36, *N* = 8; Fig. 1B). Pooling the data obtained yielded an average of 57.7±4.5 nM for the amplitude of the persistent calcium increase in hippocampal astrocytes in the presence of TTX (*n* = 52; *N* = 8). These results indicate that NH_4_^+^/NH_3_-evoked calcium increases were not dependent on action potential generation and synaptic transmitter release.

Taken together, our data show that acute perfusion with 5 mM NH_4_Cl for 30 minutes increases the intracellular calcium concentration of hippocampal astrocytes *in situ* by about 50 nM. This effect is independent from the generation of neuronal action potentials, persists as long as the NH_4_^+^/NH_3_ is present and is only partly reversible upon its removal. An additional transient increase in calcium is observed in about half the cells at the beginning of the NH_4_^+^/NH_3_ perfusion.

### Dependence of astrocyte calcium changes on NH_4_^+^/NH_3_-concentrations

Our experiments have shown that perfusion with 5 mM NH_4_^+^/NH_3_ causes a significant increase in the intracellular calcium of astrocytes. While such high NH_4_^+^/NH_3_ concentrations have been reported from animal experiments following acute liver failure [17], the increase in brain NH_4_^+^/NH_3_ will certainly not be instantaneous.

**Figure 2:**
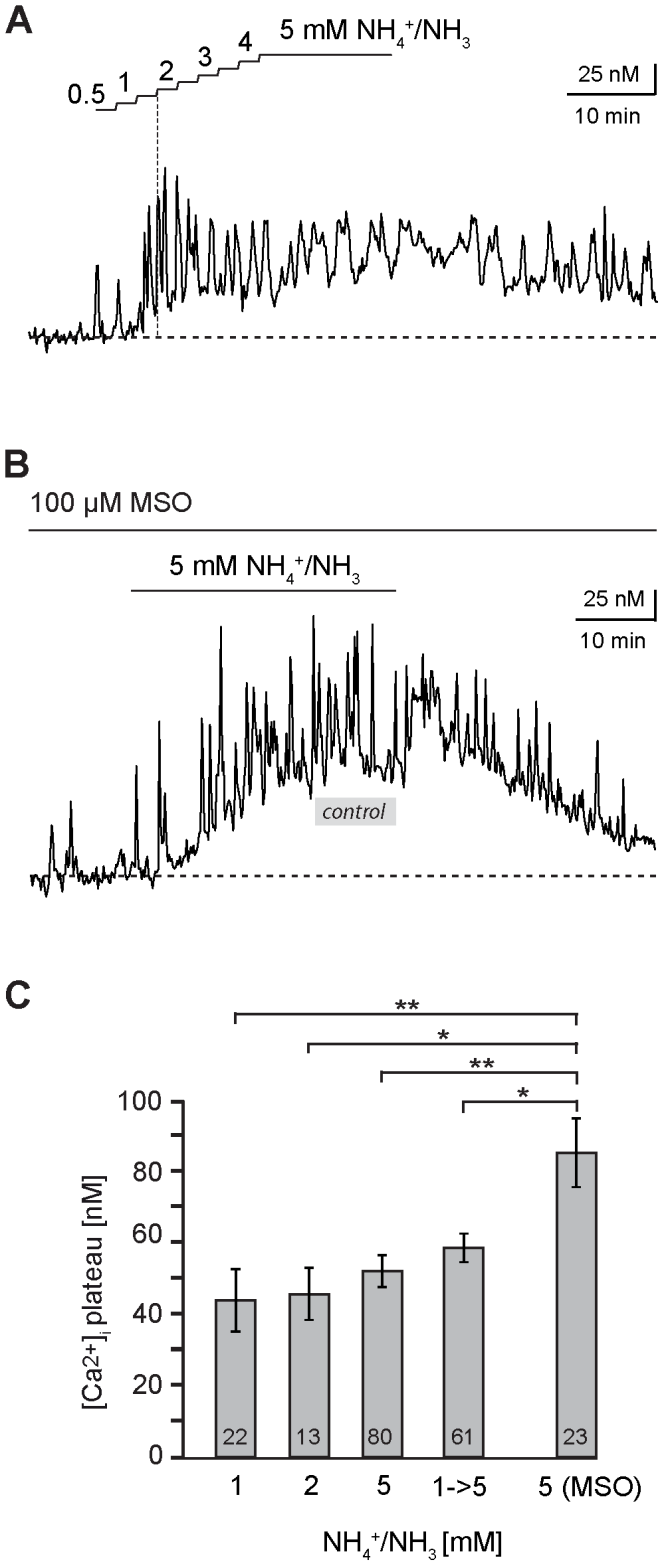
Dependence of astrocyte calcium changes on NH_4_^+^/NH_3_-concentrations. **(A)** Calcium changes in an hippocampal astrocyte evoked by stepwise increases in the NH_4_^+^/NH_3_ concentration by 0.5 mM for 2 minutes, starting from nominally 0 mM up to a final concentration of 5 mM which was then maintained for another 15 minutes. **(B)** Influence of methioninesulfoximine (MSO, 100 µM) on NH_4_^+^/NH_3_-induced calcium changes in a hippocampal astrocyte. The grey bar indicates the average amplitude of the sustained calcium increase evoked under control conditions in the absence of MSO (∼50 nM). **(C)** Histogram showing the mean peak amplitude ± S. E. M. of sustained calcium changes in response to 1, 2 and 5 mM NH_4_^+^/NH_3_, after stepwise increases from 0 to 5 mM NH_4_^+^/NH_3_ and in the presence of MSO and 5 mM NH_4_^+^/NH_3_. The number of cells is given within the bars. Peak amplitudes of calcium changes did not differ between the different concentrations used. They were, however, significantly larger in the presence of MSO as compared to all other conditions (*: p<0.05; **: p<0.01).

To mimic a slow increase in brain NH_4_^+^/NH_3_, we performed experiments in which the NH_4_^+^/NH_3_ concentration was increased stepwise every two minutes by 0.5 mM starting from nominally 0 mM up to a final concentration of 5 mM, which was then maintained for another 15 minutes (Fig. 2A). In contrast to the experiments, in which NH_4_^+^/NH_3_ was directly elevated to 5 mM (cf. Fig. 1), a defined transient increase in calcium, lasting several minutes, was not apparent at the onset of the NH_4_^+^/NH_3_ perfusion. However, a persistent increase in NH_4_^+^/NH_3_ developed (Fig. 2A), which amounted to 58.5±4.0 nM as determined in the presence of 5 mM NH_4_^+^/NH_3_, a value which is not significantly different to the one induced by direct application of 5 mM NH_4_^+^/NH_3_ (*n* = 61; *N* = 9; Fig. 2 C).

A clear increase in astrocyte calcium levels was already apparent in response to short-term application of 1–2 mM NH_4_^+^/NH_3_ (Fig. 2A). Therefore, we also studied the effect of a perfusion with 1 and 2 mM NH_4_^+^/NH_3_ only. While a transient initial increase in intracellular calcium was neither seen at 1 nor at 2 mM NH_4_^+^/NH_3_, both concentrations induced a persistent increase (1 mM: increase by 43.8±8.8 nM, *n* = 22; *N* = 4; 2 mM: increase by 45.6±7.3 nM; *n* = 13; *N* = 4; Fig. 2C). The mean values of the persistent NH_4_^+^/NH_3_-induced calcium increases were not different between the three concentrations studied (1, 2 and 5 mM; Fig. 2C).

Astrocytes metabolize NH_4_^+^/NH_3_ by the enzyme glutamine synthetase, a reaction which results in the generation of glutamine from glutamate and which plays a central role in the glutamate/glutamine cycle [9]. Glutamine synthetase activity may lower the effective intracellular NH_4_^+^ concentration and thereby reduce the effects of NH_4_^+^/NH_3_ perfusion on the intracellular calcium of astrocytes. To test this hypothesis, we applied methioninesulfoximine (MSO, 100 µM), an inhibitor of glutamine synthetase. In the presence of MSO, perfusion with 5 mM NH_4_^+^/NH_3_ induced a transient peak in 53% of investigated astrocytes, the time course and amplitude of which was not different from that without MSO (*n* = 23, *N* = 3). In contrast, the amplitude of the persistent calcium increase was increased by 64% as compared to the control (85.2±9.6 nM; *n* = 23, *N* = 3; Fig. 2B, C).

Taken together, these experiments show that NH_4_^+^/NH_3_ results in an elevation of astrocyte calcium already at concentrations of 1 mM. When NH_4_^+^/NH_3_ is not increased instantly, but slowly up to a concentration of 5 mM, clear biphasic responses are not observed. Instead, monophasic responses, represented by a relatively slow increase in astrocyte calcium to a stable plateau, prevail. Furthermore, inhibition of glutamine synthetase results in significantly larger increases in calcium in response to NH_4_^+^/NH_3_, indicating that this enzyme reduces the effective NH_4_^+^/NH_3_ concentration in astrocytes under normal conditions.

### NH_4_^+^-induced changes in astrocyte calcium in neocortex and cerebellum

**Figure 3:**
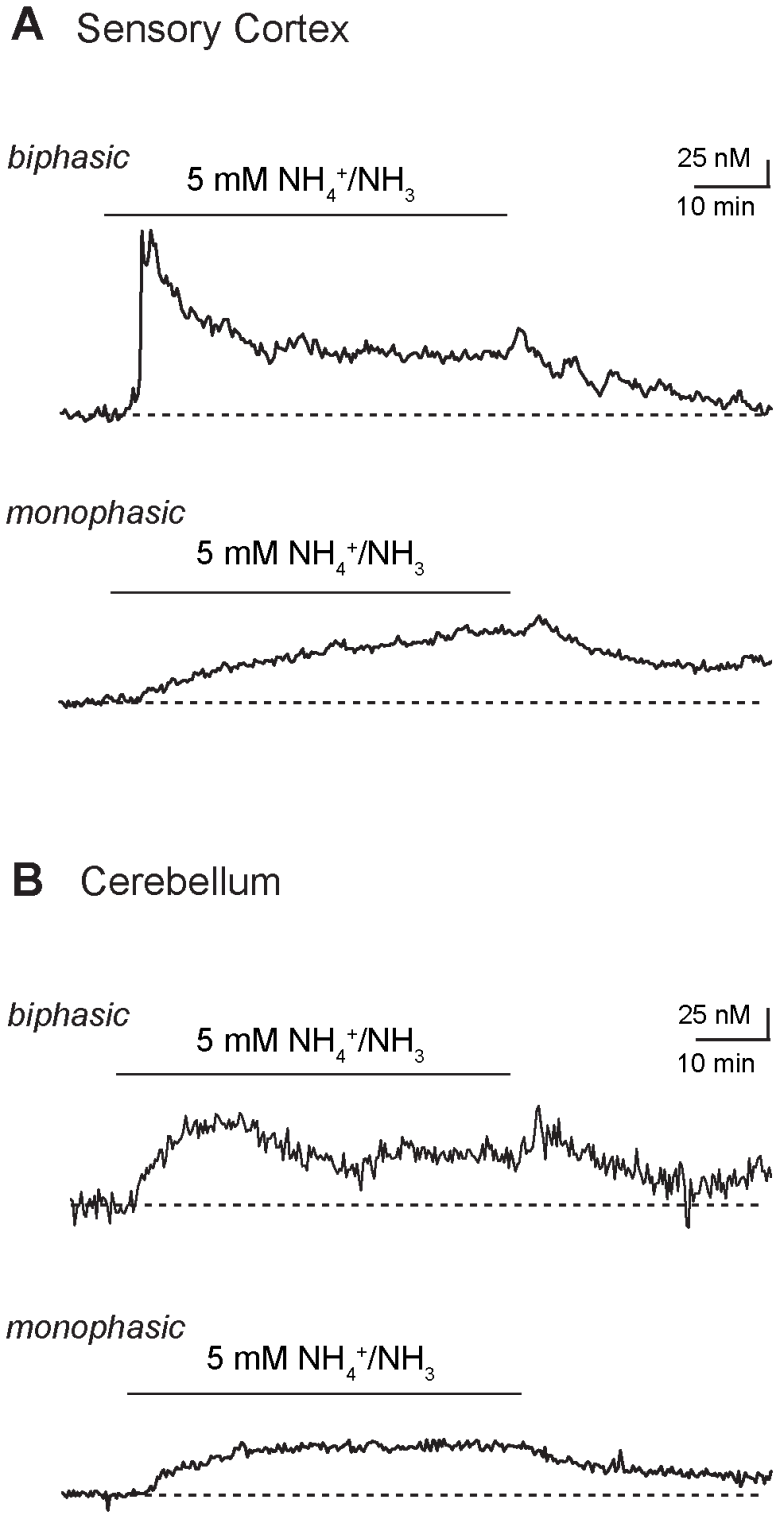
NH_4_^+^-induced changes in neocortex and cerebellum. **(A)** Calcium changes evoked by bath perfusion with 5 mM NH_4_^+^/NH_3_ for 30 minutes (indicated by bar) in two different SR101-positive astrocytes of the sensory cortex. Upper trace: biphasic response, consisting of a transient elevation followed by a plateau phase. Lower trace: monophasic response with a plateau phase only. **(B)** Calcium changes evoked by bath perfusion with 5 mM NH_4_^+^/NH_3_ for 30 minutes (indicated by bar) in two different Bergmann glial cells of the cerebellar cortex. Upper trace: biphasic response, consisting of a transient elevation followed by a plateau phase. Lower trace: monophasic response with a plateau phase only.

To study if NH_4_^+^/NH_3_-induced changes in intracellular calcium are restricted to astrocytes of the hippocampus, we prepared acute slice preparations from three different regions of the neocortex (primary motor cortex: *n* = 143, *N* = 10; somatosensory cortex: *n* = 41, *N* = 7; barrel cortex: *n* = 16, *N* = 3) as well as from the cerebellar cortex (*n* = 25, *N* = 5). In each of these investigated regions, bath perfusion with NH_4_^+^/NH_3_ in the presence of TTX elicited biphasic or monophasic elevations in astrocyte calcium (Fig. 3A, B) as observed before in the hippocampus, albeit with partly different incidence and absolute amplitudes.

Compared to astrocytes of the hippocampus, the proportion of cells responding with a biphasic signal was similar in the sensory cortex (46%), but significantly lower in the motor cortex (15%), barrel cortex (25%) and in Bergman glial cells of the cerebellum (12%). In biphasic cells, the peak amplitude of the initial transient signal was significantly smaller in barrel cortex and cerebellum as compared to hippocampus (amplitude of transients in motor cortex: 83.5±13.0 nM (*n* = 22); sensory cortex: 86.2±12.1 nM (*n* = 19); barrel cortex: 43.3±6.2 (*n* = 4); cerebellar Bergmann glia: 62.2±13.1 nM (*n* = 3)). The average amplitude of the plateau phase of mono- and biphasic cells was significantly smaller in the sensory cortex, barrel cortex and cerebellum as compared to the hippocampus (motor cortex: 50.3±2.6 nM; sensory cortex: 45.1±3.1 nM; barrel cortex: 34.5±3.5 nM; cerebellum: 37.7±3.1 nM). As frequently observed in the hippocampus, calcium levels did not fully recover to baseline levels in the majority of cells and 30 minutes after removal of NH_4_^+^/NH_3_, calcium concentration was still increased by 20–40 nM (Fig. 3A, B).

Taken together, these experiments show that perfusion with 5 mM NH_4_^+^/NH_3_ elicits changes in the intracellular calcium concentration in SR101-positive astrocytes in several regions of the neocortex as well as in cerebellar Bergmann glial cells. Despite some region-specific heterogeneity concerning the relative incidence of the response types and the absolute amplitude of calcium changes, these changes are qualitatively similar to those of the hippocampus, suggesting that they represent a more general phenomenon.

### Interrelationship of NH_4_^+^/NH_3_-induced calcium changes with pH and sodium changes

**Figure 4:**
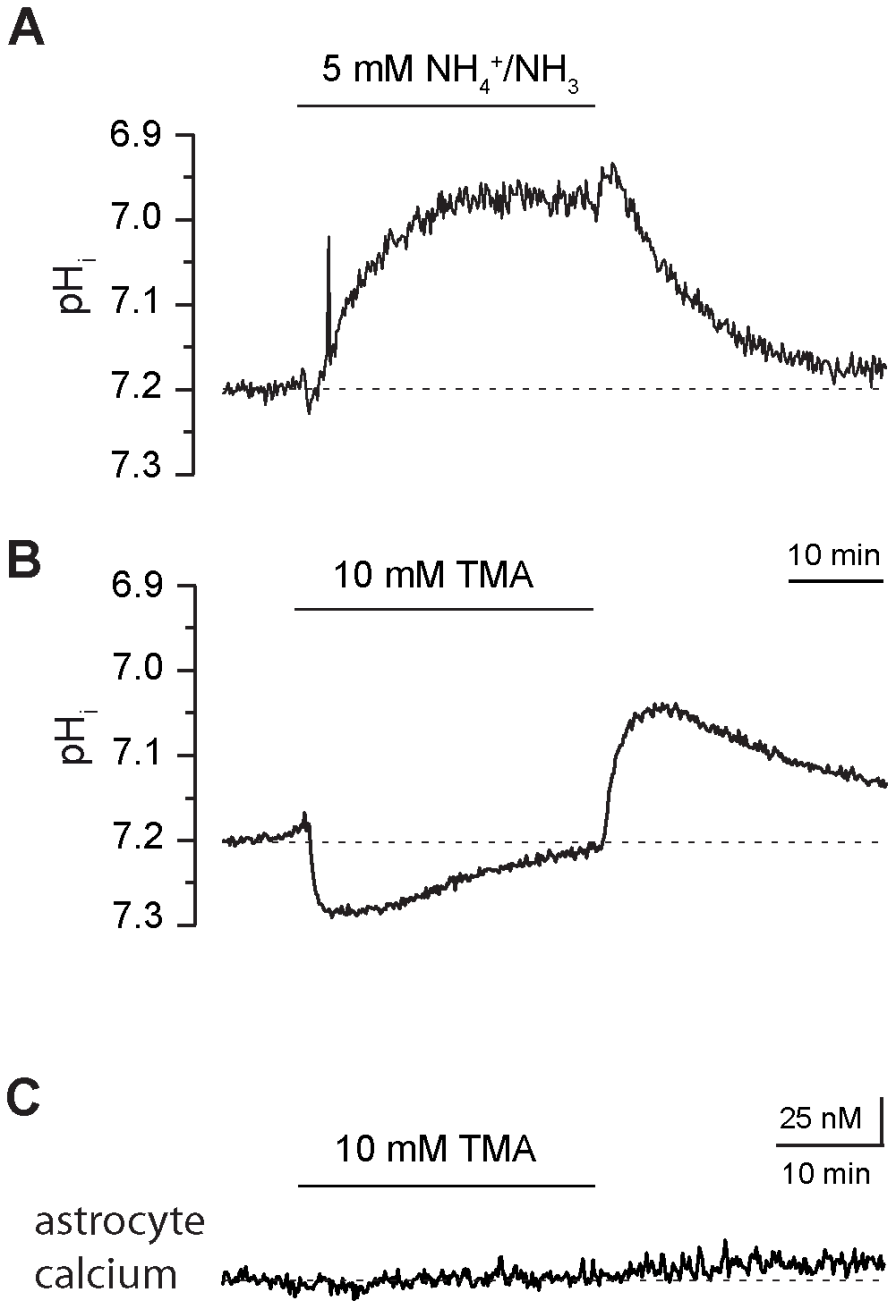
Interrelationship between NH_4_^+^-induced changes in calcium and pH. **(A)** Changes in intracellular pH (pH_i_) evoked by bath perfusion with 5 mM NH_4_^+^/NH_3_ for 30 minutes (indicated by bar) in a SR101-positive hippocampal astrocyte. **(B)** Changes in a hippocampal astrocyte evoked by perfusion with the weak base trimethylamine (TMA, 10 mM) for 30 minutes (indicated by bar). Note the pronounced alkalinization, which is followed by an acidification upon removal of TMA. **(C)** Trace showing intracellular calcium in a hippocampal astrocyte and the influence of perfusion with TMA (10 mM). Note that TMA does not evoke any changes in astrocyte calcium.

To study if the NH_4_^+^/NH_3_-induced calcium increase might be causally linked to an intracellular alkalinization as resported earlier from astrocytes in culture [33], we employed ratiometric imaging with the pH-sensitive dye BCECF in hippocampal astrocytes. As reported before (e. g. [28]), we found that perfusion with 5 mM NH_4_^+^/NH_3_ caused a transient alkalinization by about 0.04 pH units, which lasted 1–2 minutes and was followed by a sustained acidification by 0.18 pH units (*n* = 17, *N* = 4; Fig. 4A). Moreover, we tested the effect of the weak base trimethylamine (TMA), another established tool to manipulate intracellular pH. As expected (e. g. [28]), bath perfusion with TMA (10 mM) caused an alkalinisation. The alkalinisation reached its peak of about 0.1 pH units after 10 minutes and then slowly declined in the sustained presence of TMA (*n* = 18, *N* = 3; Fig. 4A). Upon removal of TMA, a pronounced rebound acidification was observed, after which intracellular pH slowly recovered back to its initial baseline (Fig. 4B). In contrast to the results obtained in cultured cortical astrocytes [33], the pronounced alkalinization induced by TMA was never accompanied by any change in intracellular calcium (*n* = 30, *N* = 4; Fig. 4C).

In the further course of studying the mechanisms by which NH_4_^+^/NH_3_-influences intracellular calcium in hippocampal astrocytes, we concentrated on the plateau phase because it is evoked in every responding cell and is the dominating signal also with stepwise increases in NH_4_^+^/NH_3_. It should be noted, however, that none of the manipulations performed and described in the following altered one of the phases specifically or separately, but generally influenced both the transient and plateau phase in a similar manner.

In an earlier work, we have shown that NH_4_^+^ is transported into astrocytes through NKCC1 and thereby causes a substantial increase in the intracellular sodium concentration [28], [29]. Intracellular sodium and calcium homeostasis in astrocytes are interconnected through the sodium/calcium exchanger (NCX), which plays a role in calcium extrusion [47], [48]. Because NCX works close to its reversal potential, it can, however, also switch to reverse mode upon increases in intracellular sodium and mediate the uptake of calcium [49]. Application of KB-R7943 (10 µM), which blocks the reverse mode of NCX [49], did not alter NH_4_^+^/NH_3_-induced calcium increases in astrocytes (*n* = 24, *N* = 3), indicating that this mechanism is not involved in their generation. This notion was supported by the fact that bumetanide (100 µM), an inhibitor of NKCC1, which omits NH_4_^+^/NH_3_-induced sodium elevations in astrocytes [28], [29], also did not did not significantly reduce the NH_4_^+^/NH_3_-induced calcium increase (*n* = 13, *N* = 3).

In summary, these results demonstrate that neither the transient, nor the persistent NH_4_^+^/NH_3_-induced calcium increase in hippocampal astrocytes *in situ* are causally linked to an intracellular alkalinization. Similarly, activation of NKCC1, subsequent intracellular sodium accumulation and reversal of NCX do not play a role in the generation of NH_4_^+^/NH_3_-induced calcium increases in this preparation.

### Involvement of glutamate receptors in NH_4_^+^/NH_3_-induced calcium changes

There is experimental evidence that hyperammonemic conditions provoke a release of glutamate, which then acts on glutamate receptors on neurons and glial cells [33], [50], [51]. Such a mechanism might be involved in the generation of NH_4_^+^/NH_3_-induced calcium elevations in astrocytes. To test this hypothesis, we tested the effect of blockers of ionotropic and metabotropic glutamate receptors on the calcium increase induced by NH_4_^+^/NH_3_.

**Figure 5:**
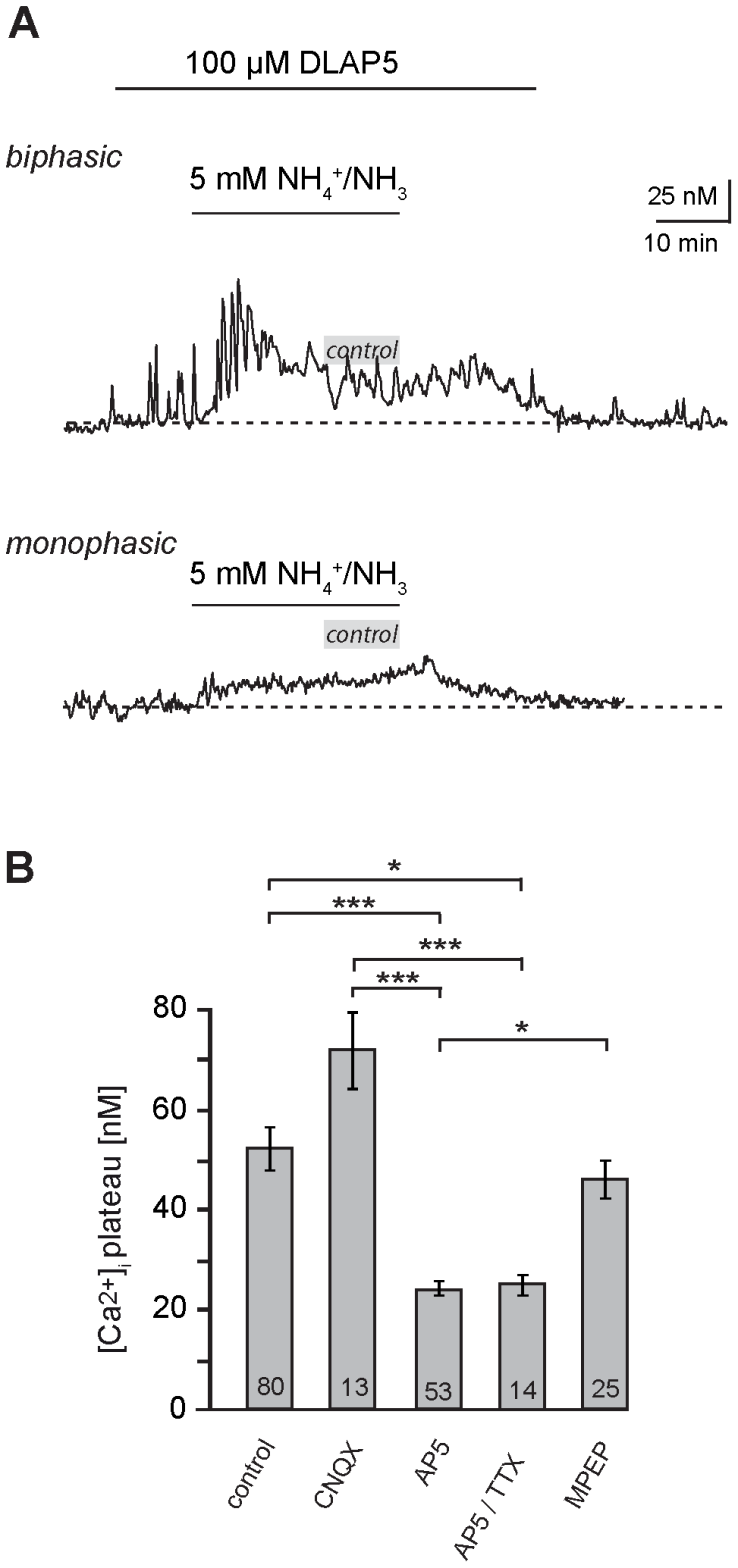
Involvement of glutamate receptors in NH_4_^+^/NH_3_-induced calcium changes. **(A)** Influence of the NMDA-receptor blocker DL-AP5 (100 µM, indicated by bar) on NH_4_^+^/NH_3_-induced calcium changes in a biphasic (upper trace) and a monophasic (lower trace) hippocampal astrocyte. The grey bars indicate the average amplitude of the sustained calcium increase evoked under control conditions in the absence of DLAP5 (∼50 nM). **(B)** Histogram showing the mean peak amplitude ± S. E. M. of sustained calcium changes in response to NH_4_^+^/NH_3_ in the control, and in the presence of the glutamate receptors blocker CNQX, DLAP5 (AP5), DLAP5 in the presence of TTX, or MPEP. The number of cells is given within the bars. AP5 and AP5/TTX reduce the amplitude of calcium increases significantly as compared to the control (***: p<0.001; *: p<0.05).

Bath application of CNQX (100 µM), a blocker of ionotropic AMPA-receptor channels, did not significantly alter the time course nor the amplitude of the NH_4_^+^/NH_3_-induced calcium increase in astrocytes (*n* = 13, *N* = 3; Fig. 5B). In the presence of DL-AP5 (100 µM), an inhibitor of ionotropic NMDA-receptors, however, the amplitude of the calcium elevation in hippocampal astrocytes was reduced by 54% (23.9±1.3 nM; *n* = 53; *N* = 7; Fig. 5A, B). Combined application of DL-AP5 with TTX (500 µM) did not reduce the amplitude of calcium elevations further, again indicating that the opening of voltage-gated sodium channels and action potential generation as well as action-potential induced transmitter release were not involved in their generation (25.1±2.1 nM; *n* = 14, *N* = 2; Fig. 5B). Finally, we studied the dependence of NH_4_^+^/NH_3_-induced calcium signals on activation of metabotropic glutamate receptors (mGluR). Because astrocytes mainly express mGluR5, we applied 2-methyl-6-(phenylethynyl)-pyridine (MPEP, 25 µM) an antagonist of these receptors. MPEP did not significantly affect the amplitude of the evoked calcium increase (*n* = 25, *N* = 5; Fig. 5B).

Taken together, these results indicate that neither AMPA receptors nor mGluR5 contribute to the generation of NH_4_^+^/NH_3_-induced calcium signals in hippocampal astrocytes *in situ*. Instead our data suggest an involvement of NMDA receptor activation, an effect which is independent from action-potential mediated transmitter release.

### Involvement of intracellular stores in NH_4_^+^/NH_3_-induced calcium changes

**Figure 6:**
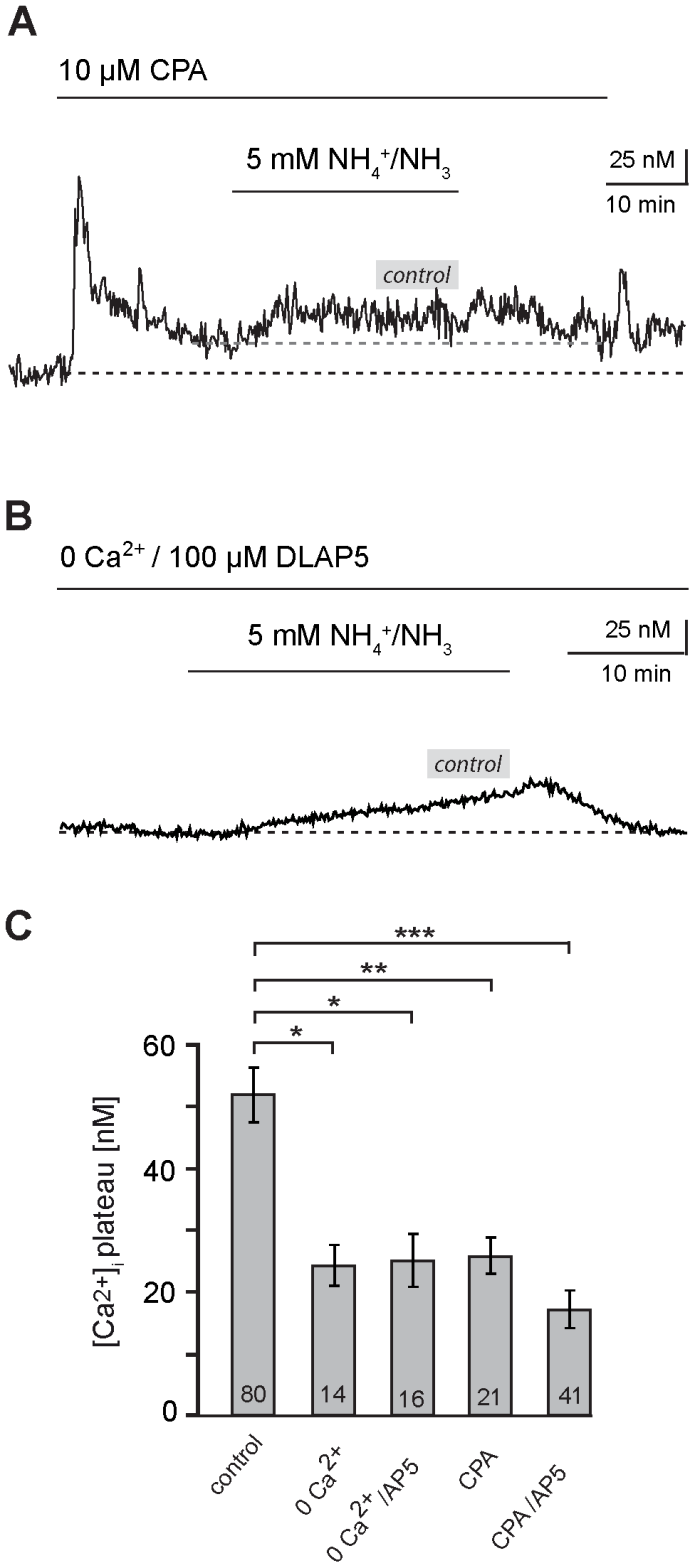
Involvement of calcium influx and intracellular stores in NH_4_^+^/NH_3_-induced calcium changes. **(A)** Influence of combined removal of extracellular calcium with the NMDA-receptor blocker DLAP5 (“0Ca^2+^/100 µM DLAP5”; indicated by bar) on NH_4_^+^/NH_3_-induced calcium changes in a hippocampal astrocyte. Note the decrease in calcium upon removal of calcium. **(B)** Influence of the SERCA blocker CPA (10 µM, indicated by bar) on NH_4_^+^/NH_3_-induced calcium changes in a hippocampal astrocyte. Note the biphasic calcium elevation induced at the onset of CPA perfusion. **(A)**, **(B)** The grey bars indicate the average amplitude of the sustained calcium increase evoked under control conditions in the absence of blockers (∼50 nM) **(C)** Histogram showing the mean peak amplitude ± S. E. M. of sustained calcium changes in response to NH_4_^+^/NH_3_ in the control, in the nominal absence of extracellular calcium, in the absence of extracellular calcium combined DLAP5, in the presence of CPA and in the combined presence of CPA and DLAP5. The number of cells is given within the bars; all manipulations result in significantly smaller changes in calcium as compared to the control (***: p<0.001; **: p<0.01; *: p<0.05).

To further study the pathway by which NH_4_^+^/NH_3_ causes an increase in intracellular calcium, we performed experiments in which slices were perfused with nominally calcium-free saline. In the absence of extracellular calcium, the mean amplitude of NH_4_^+^/NH_3_-induced increases in astrocyte calcium was reduced by 54% (24.0±3.0 nM; *n* = 14, *N* = 3; Fig. 6C), a value, which was not significantly altered if NMDA receptors were additionally blocked by DL-AP5 (24.9±4.3 nM; *n* = 16, *N* = 3; Fig. 6A, C). The latter result indicates that the involvement of NMDA receptor activation in the generation of NH_4_^+^/NH_3_-induced calcium changes in astrocytes requires extracellular calcium.

Moreover, we tested the involvement of calcium release from intracellular stores. To this end, we applied cyclopiazonic acid (CPA; 10 µM), which inhibits the Ca^2+^-ATPase of the endoplasmic reticulum (SERCA pump; [52]) and thereby causes a depletion of these stores. As shown in earlier studies, application of CPA itself caused a biphasic calcium increase by about 40 nM (37.1±3.2; *n* = 21, *N* = 4; Fig. 6B) due to leakage of calcium from the ER [44], [53]. Under these conditions, the amplitude of the NH_4_^+^/NH_3_-induced calcium changes was reduced by about 50% (25.8±2.9 nM; *n* = 21, *N* = 4; Fig. 6B, C). During combined application of DL-AP5 and CPA, the NH_4_^+^/NH_3_-induced calcium increase was reduced by about 67% (17.1±3.0 nM; *n* = 41, *N* = 7; Fig. 6C), which is not significantly different to the reduction evoked by CPA alone, but significantly different to the reduction evoked by application of DL-AP5 alone (cf. Fig. 5B).

Our data thus suggest that major mechanisms in the generation of NH_4_^+^/NH_3_-induced calcium increases in hippocampal astrocytes involve activation of NMDA receptors and influx of calcium from the extracellular space as well as release of calcium from intracellular stores.

## Discussion

In the present study, we show that bath perfusion with NH_4_^+^/NH_3_ causes sustained increases in the intracellular calcium concentration of SR101-positive astrocytes of the hippocampus, different regions of the neocortex, as well as in Bergmann glial cells. NH_4_^+^/NH_3_-induced calcium elevations are augmented following inhibition of glutamine synthetase and are not mimicked by intracellular pH changes evoked by the weak base TMA. Neither TTX, a blocker of voltage-gated sodium channels, nor bumetanide, a blocker of NKCC1, nor KB-R793, a blocker of reverse NCX, diminish the NH_4_^+^/NH_3_-induced calcium increases. The same is true for blockers of ionotropic AMPA or metabotropic GluR5 receptors. In contrast to this, perfusion with DL-AP5, which blocks NMDA receptors, or depletion of intracellular calcium stores by CPA significantly dampen NH_4_^+^/NH_3_-induced calcium changes in astrocytes.

### NH_4_^+^/NH_3_ affects astrocyte calcium in different brain regions

Based on our calibration procedure, we determined a baseline calcium concentration of about 155 nM in hippocampal astrocytes, a value which is well within the range of those reported by others (e. g. [54], see also Table 1 in [55]). Perfusion with 1–5 mM NH_4_^+^/NH_3_ for 30 minutes caused a sustained increase in the intracellular calcium concentration. Surprisingly, the absolute amplitudes of calcium increases were similar in response to 1, 2, and 5 mM NH_4_^+^/NH_3_. This result might at first seem surprising. However, after blocking glutamine synthesis with MSO, we found a strong increase of the amplitude of the persistent calcium increase in response to 5 mM NH_4_^+^/NH_3_, indicating that much of the ammonium applied by bath perfusion is quickly detoxified by astrocytes. Thus, as argued below, glutamine synthetase activity apparently significantly influences the effective intracellular NH_4_^+^ concentration and which might have obstructed differences in the NH_4_^+^/NH_3_ concentration present in the perfusion saline.

The ammonium-induced increase in astrocyte calcium amounted to about 50 nM. This value is quite similar to that reported in earlier studies performed on cultured mouse cortical astrocytes [33] and on cultured rat astrocytes [32]. Noteworthy, our data show that even large changes in intracellular pH as those produced by TMA were not accompanied by any detectable change in the ratio of Fura-2. This shows that ammonium-induced changes in pH are not the primary cause for the observed changes in the Fura-2 ratio, and that the latter are neither a consequence of the pH-sensitivity of the dye, nor a consequence of real changes in intracellular pH.

NH_4_^+^/NH_3_-evoked calcium increases were not only consistently observed in hippocampal astrocytes, but also in astrocytes in different areas of the neocortex and in Bergmann glial cells. The disturbance of glial calcium by NH_4_^+^/NH_3_ therefore, seems to be a widespread, if not general, phenomenon in the brain. While, as mentioned above, studies in primary cell culture also described an increase in intracellular calcium upon NH_4_^+^/NH_3_ application [32], [33], the time course and duration of evoked calcium changes differed between the former two studies. In cultured mouse astrocytes, only an initial, short transient was observed [33], cultured rat astrocytes, however, underwent a slow, but sustained increase in calcium [32]. In the present study, astrocytes *in situ* showed either a monophasic, sustained increase as reported from rat astrocytes [32] or a biphasic signal, where the sustained increase was preceded by a transient increase in calcium at the beginning of the NH_4_^+^/NH_3_ perfusion. This initial transient was similar to the signal observed in cultured mouse astrocytes under comparable experimental conditions [33].

The two response types occurred in every brain region investigated upon changing from nominally NH_4_^+^/NH_3_-free saline to saline containing 5 mM NH_4_^+^/NH_3_. While NH_4_^+^/NH_3_ concentrations have been reported to rise to levels of more than 5 mM in animal models of acute liver failure [17], the increase in brain NH_4_^+^/NH_3_ concentration will certainly not be instantaneous. To mimic this situation, we performed experiments in which the NH_4_^+^/NH_3_ concentration was increased from nominally 0 mM to 5 mM in small steps of 0.5 mM, each lasting 2 minutes. As observed with application of 1 or 2 mM NH_4_^+^/NH_3_ only, a pronounced initial transient increase in calcium was absent under this condition, while the sustained calcium signal persisted. This suggests that the sustained monophasic calcium elevation in response to ammonia observed in the vast majority of astrocytes investigated might also be the dominating signal evoked in the intact brain during HE.

### Glutamine synthetase protects against NH_4_^+^/NH_3_-induced calcium dysbalance

Glutamine synthetase, which catalyzes the generation of glutamine from glutamate and NH_4_^+^, is a glial-specific key enzyme and central for the detoxification of NH_4_^+^ in the brain [5]. Increased levels of NH_4_^+^/NH_3_ will affect this reaction and glutamine concentrations increased six-fold in an animal model of acute liver failure [17]. It has been proposed that the elevation of glutamine levels is causally linked to several effects triggered by hyperammonemia including the generation of reactive oxygen species and oxidative stress as well as astrocyte swelling [12], [56], [57]. This is in line with observations showing that inhibition of glutamine synthetase by MSO ameliorates astrocyte swelling and brain edema in hyperammonemic conditions [24], [58].

In the present study, inhibition of glutamine synthetase by MSO did not reduce, but significantly augment the NH_4_^+^/NH_3_-induced calcium increases in astrocytes. This shows that glutamine synthetase activity and glutamine accumulation are not a primary cause of calcium dysbalance. Instead, our results indicate that glutamine synthetase dampens the harmful effects of acute NH_4_^+^/NH_3_ intoxication by reducing its effective concentration. In addition, NH_4_^+^/NH_3_ might be metabolized not only via glutamine synthetase, but by the action of glutamate dehydrogenase (GDH) and alanine aminotransferase (ALAT) which results in alanine formation [59]. These enzymes, might, therefore, additionally protect the tissue from harmful effects of hyperammonemia.

### NH_4_^+^/NH_3_-induced calcium dysbalance is independent from concomitant changes in intracellular pH and sodium

NH_4_^+^/NH_3_-induced astrocyte calcium signals persisted in the presence of TTX, which blocks voltage-dependent sodium channels and action potential generation by neurons. This demonstrates that they are not primarily related to action-potential-induced transmitter release and neuronal network activity in the slice preparation. Thus, NH_4_^+^/NH_3_ might act on astrocytes directly. The passage of NH_4_^+^/NH_3_ across cell membranes results in stereotypical changes in intracellular pH, which consist of an intracellular alkalinisation followed by pronounced intracellular acidification that develops in the continued presence of NH_4_^+^/NH_3_ [27], [28]. It was suggested earlier in cultured cerebral astrocytes, that the initial alkalinisation causes release of calcium from intracellular stores, thereby resulting in the generation of a NH_4_^+^/NH_3_-induced transient calcium elevation [33]. Our data presented here, are in contrast to this and strongly suggest that the NH_4_^+^/NH_3_-induced calcium increases in astrocytes *in situ* are not directly causally linked to changes in their intracellular pH.

A basic difference between the results presented here and those of C. Rose and co-workers [33] is the pH change induced by ammonium. The latter reported an alkalinization in response to ammonium, whereas we found a very brief and small alkalinization only that was followed by a large and sustained acidification, a result that confirmed our earlier study in this preparation [28]. This difference is likely to be explained by the different model systems used. C. Rose performed his study in primary cultures of cortical astrocytes and cells were used between days 11–15 in culture, that is at a relatively early stage. In our own culture work performed earlier [29], we reported that between 9–16 days *in vitro*, astrocytes undergo a pure alkalinization in response to ammonium application, similar to what C. Rose reported. In contrast to this, however, astrocytes cultured for 20–34 days only showed a brief alkalinization followed by a prolonged acidification with ammonium [29]. This age-dependent difference was mainly due to the strong up-regulation of NKCC1 found after two weeks in culture, also demonstrated in our former work [29]. Thus, one can assume that the cultured astrocytes investigated by C. Rose were in less differentiated state than those investigated in acute tissue slices in the present study.

In the study by C. Rose [33], the ammonium-induced transient alkalinization induced a transient increase in intracellular calcium in cultured astrocytes due to the release of calcium from intracellular stores, an effect, which was mimicked by the weak base TMA. As expected, TMA evoked a pronounced biphasic shift in the intracellular pH also in our hands. However, we found that neither the pronounced and sustained alkalinization, nor the subsequent acidification induced by the weak base TMA, were accompanied by intracellular calcium signals. The reason for this discrepancy is unclear. In analogy to the argumentation presented above, it could be speculated that cultured astrocytes that are not fully differentiated yet, differ from more mature astrocytes as those found in acute tissue slices after the second postnatal week in their capability to buffer calcium, i. g. by a lower expression of calcium-binding proteins. Alternatively, the mechanisms which cause a pH-dependent release of calcium from intracellular stores might differ between astrocytes in culture and those in the intact tissue.

We have shown earlier that NH_4_^+^ activates NKCC1 in astrocytes, resulting in a substantial increase in the intracellular sodium concentration [28], [29]. NKCC1 plays a central role in the swelling of astrocytes [60], and has also been linked to NH_4_^+^/NH_3_-induced cell swelling [61]. Application of bumetanide, which efficiently blocks NKCC1 and related sodium elevations in astrocytes in hippocampal slice preparations [28], did not reduce NH_4_^+^/NH_3_-induced calcium signals in the present study. Intracellular sodium and calcium homeostasis in astrocytes are interconnected through the action of NCX, which works close to its equilibrium potential under resting conditions[47], [49]. While there is evidence that NCX can reverse upon increases in intracellular sodium [49], blocking its reverse mode operation by KB-R793 did not reduce NH_4_^+^/NH_3_-induced calcium elevations. Thus, NH_4_^+^/NH_3_-induced calcium signals in astrocytes are not caused by activation of NKCC1, nor by subsequent intracellular sodium accumulation and reversed uptake of calcium through the NCX.

### Involvement of glutamatergic transmission and store-mediated release of calcium

NH_4_^+^/NH_3_-induced calcium signals in astrocytes persisted in the presence of TTX, and are thus independent from action-potential-induced release of transmitters from neurons (see above). However, there is ample evidence for a disturbance of glutamatergic neurotransmission under hyperammonemic conditions [1]. We, therefore, tested the effect of blockers of different glutamate receptors. While neither blocking ionotropic AMPA receptors nor mGluR5 altered NH_4_^+^/NH_3_-induced calcium signals, their amplitude was reduced by about 50% following inhibition of NMDA receptors both in the presence and absence of TTX. Thus, activation of NMDA receptors contributes to the generation of NH_4_^+^/NH_3_-induced calcium signals in hippocampal astrocytes *in situ*.

Acute hyperammonemia has been reported to cause an over-activation of NMDA receptors which seems to play a vital role in the generation of its toxic effects [62]–[64]. In fact, blocking NMDA receptors increased the survival of animals subjected to hyperammonemia and acute liver failure [1], [9]. Our results support this notion by showing that NMDA receptor activation is also involved in the NH_4_^+^/NH_3_-induced calcium dysbalance in hippocampal astrocytes. While this is in line with earlier suggestions based on studies on cortical astrocytes [25], hippocampal astrocytes, in contrast to cortical astrocytes, are commonly regarded as being devoid of NMDA receptors [65], [66]. This suggests that the reduction of astrocyte calcium signals in the presence of NMDA receptor blockers is an indirect effect, resulting from blocking neuronal receptors. On the other hand, calcium transients induced by focal application of glutamate in astrocytes of rat hippocampal slices were shown to partly result from activation of NMDA receptors as well [67]. Thus, a direct effect of NMDA receptor inhibition on astrocytes cannot definitely be excluded in our study.

NH_4_^+^/NH_3_-induced calcium increases in response to NH_4_^+^/NH_3_ were strongly dampened in nominally calcium-free saline and blocking of NMDA receptor activation under this condition resulted in no further reduction. Again, this result is in contrast to the study performed earlier in astrocyte cultures, in which calcium signals were not influenced by removal of extracellular calcium [33]. A likely explanation for this discrepancy might be that astrocytes in culture were in a less differentiated state as argued above. Moreover, the presence of neurons and the interaction between neurons and astrocytes in our slice preparation might be another cause of this discrepancy. Our work suggests that NH_4_^+^/NH_3_-induced calcium changes are largely mediated by influx of calcium from the extracellular space and that this is the mechanism by which NMDA receptor activation contributes to their generation. However, these results have to be interpreted with caution because earlier work has established that there is a rapid depletion of intracellular stores in astrocytes in acute tissue slices in calcium-free saline [44]. Indeed, depletion of intracellular stores by CPA also reduced calcium signals by about 50%, indicating that release from intracellular stores is a second important pathway. Combined application of CPA and DL-APV diminished calcium increases by 67%, showing that both NMDA receptor activation and release of calcium from intracellular stores are the main mechanisms by which NH_4_^+^/NH_3_-induced calcium elevations in astrocytes arise.

## Conclusions

Our data establish that even moderate increases in NH_4_^+^/NH_3_ cause a persistent increase in calcium in astrocytes in acute tissue slice preparations, an observation that seems to be a widespread, if not general, phenomenon in different regions of the brain. Astrocyte calcium has been reported to be an important modulator of brain microcirculation [68]. Furthermore, calcium homeostasis and signaling in astrocytes play an important role in neuron-glia interaction and neuronal plasticity [69], [70]. The dysbalance in astrocyte calcium could thus promote alterations in synaptic plasticity as observed under hyperammonemic conditions [71]–[74] and thereby contribute to the symptoms of HE.

## Supporting Information

Data S1 Spreadsheets providing data sets as illustrated by Figures 1–6, individual data points utilized for statistical analysis as presented in the results (expressed as baseline calcium levels in nM as well as changes in calcium), as well as datasets obtained in the calibration procedure as described in the Methods section.
(XLSX)

## References

[1] Felipo V (2013) Hepatic encephalopathy: effects of liver failure on brain function. Nat Rev Neurosci 14: 851–858. 24149188 10.1038/nrn3587

[2] Felipo V, Butterworth RF (2002) Neurobiology of ammonia. Prog Neurobiol 67: 259–279. 12207972 10.1016/s0301-0082(02)00019-9

[3] Rao KV, Norenberg MD (2001) Cerebral energy metabolism in hepatic encephalopathy and hyperammonemia. Metab Brain Dis 16: 67–78. 11726090 10.1023/a:1011666612822

[4] Butterworth RF (2010) Altered glial-neuronal crosstalk: cornerstone in the pathogenesis of hepatic encephalopathy. Neurochem Int 57: 383–388. 20350577 10.1016/j.neuint.2010.03.012

[5] Martinez-Hernandez A, Bell KP, Norenberg MD (1977) Glutamine synthetase: glial localization in brain. Science 195: 1356–1358. 14400 10.1126/science.14400

[6] Lemberg A, Fernandez MA (2009) Hepatic encephalopathy, ammonia, glutamate, glutamine and oxidative stress. Ann Hepatol 8: 95–102. 19502650

[7] Bak LK, Schousboe A, Waagepetersen HS (2006) The glutamate/GABA-glutamine cycle: aspects of transport, neurotransmitter homeostasis and ammonia transfer. J Neurochem 98: 641–653. 16787421 10.1111/j.1471-4159.2006.03913.x

[8] Sergeeva OA (2013) GABAergic transmission in hepatic encephalopathy. Arch Biochem Biophys 536: 122–130. 23624382 10.1016/j.abb.2013.04.005

[9] Cauli O, Rodrigo R, Llansola M, Montoliu C, Monfort P, et al (2009) Glutamatergic and gabaergic neurotransmission and neuronal circuits in hepatic encephalopathy. Metab Brain Dis 24: 69–80. 19085094 10.1007/s11011-008-9115-4

[10] Palomero-Gallagher N, Zilles K (2013) Neurotransmitter receptor alterations in hepatic encephalopathy: a review. Arch Biochem Biophys 536: 109–121. 23466244 10.1016/j.abb.2013.02.010

[11] Albrecht J, Zielinska M, Norenberg MD (2010) Glutamine as a mediator of ammonia neurotoxicity: A critical appraisal. Biochem Pharmacol 80: 1303–1308. 20654582 10.1016/j.bcp.2010.07.024 PMC4714775

[12] Desjardins P, Du T, Jiang W, Peng L, Butterworth RF (2012) Pathogenesis of hepatic encephalopathy and brain edema in acute liver failure: role of glutamine redefined. Neurochem Int 60: 690–696. 22382077 10.1016/j.neuint.2012.02.001

[13] Hindfelt B, Plum F, Duffy TE (1977) Effect of acute ammonia intoxication on cerebral metabolism in rats with portacaval shunts. J Clin Invest 59: 386–396. 838855 10.1172/JCI108651 PMC333373

[14] Lavoie J, Giguere JF, Layrargues GP, Butterworth RF (1987) Amino acid changes in autopsied brain tissue from cirrhotic patients with hepatic encephalopathy. J Neurochem 49: 692–697. 2886551 10.1111/j.1471-4159.1987.tb00949.x

[15] Kosenko E, Kaminsky YG, Felipo V, Minana MD, Grisolia S (1993) Chronic hyperammonemia prevents changes in brain energy and ammonia metabolites induced by acute ammonium intoxication. Biochim Biophys Acta 1180: 321–326. 8422438 10.1016/0925-4439(93)90057-8

[16] Mans AM, Saunders SJ, Kirsch RE, Biebuyck JF (1979) Correlation of plasma and brain amino acid and putative neurotransmitter alterations during acute hepatic coma in the rat. J Neurochem 32: 285–292. 33231 10.1111/j.1471-4159.1979.tb00350.x

[17] Swain M, Butterworth RF, Blei AT (1992) Ammonia and related amino acids in the pathogenesis of brain edema in acute ischemic liver failure in rats. Hepatology 15: 449–453. 1544626 10.1002/hep.1840150316

[18] Watanabe A, Takei N, Higashi T, Shiota T, Nakatsukasa H, et al (1984) Glutamic acid and glutamine levels in serum and cerebrospinal fluid in hepatic encephalopathy. Biochem Med 32: 225–231. 6150706 10.1016/0006-2944(84)90076-0

[19] Bender AS, Norenberg MD (1998) Effect of benzodiazepines and neurosteroids on ammonia-induced swelling in cultured astrocytes. J Neurosci Res 54: 673–680. 9843158 10.1002/(SICI)1097-4547(19981201)54:5<673::AID-JNR12>3.0.CO;2-P

[20] Blei AT, Olafsson S, Therrien G, Butterworth RF (1994) Ammonia-induced brain edema and intracranial hypertension in rats after portacaval anastomosis. Hepatology 19: 1437–1444. 8188174

[21] Ganz R, Swain M, Traber P, DalCanto M, Butterworth RF, et al (1989) Ammonia-induced swelling of rat cerebral cortical slices: implications for the pathogenesis of brain edema in acute hepatic failure. Metab Brain Dis 4: 213–223. 2796874 10.1007/BF01000297

[22] Swain MS, Blei AT, Butterworth RF, Kraig RP (1991) Intracellular pH rises and astrocytes swell after portacaval anastomosis in rats. Am J Physiol 261: R1491–1496. 1750572 10.1152/ajpregu.1991.261.6.R1491 PMC2807133

[23] Traber PG, Dal Canto M, Ganger DR, Blei AT (1987) Electron microscopic evaluation of brain edema in rabbits with galactosamine-induced fulminant hepatic failure: ultrastructure and integrity of the blood-brain barrier. Hepatology 7: 1272–1277. 3679092 10.1002/hep.1840070616

[24] Vaquero J, Butterworth RF (2007) Mechanisms of brain edema in acute liver failure and impact of novel therapeutic interventions. Neurol Res 29: 683–690. 18173908 10.1179/016164107X240099

[25] Gorg B, Schliess F, Haussinger D (2013) Osmotic and oxidative/nitrosative stress in ammonia toxicity and hepatic encephalopathy. Arch Biochem Biophys 536: 158–163. 23567841 10.1016/j.abb.2013.03.010

[26] Nagaraja TN, Brookes N (1998) Intracellular acidification induced by passive and active transport of ammonium ions in astrocytes. Am J Physiol 274: C883–891. 9575784 10.1152/ajpcell.1998.274.4.C883

[27] Thomas RC (1984) Experimental displacement of intracellular pH and the mechanism of its subsequent recovery. J Physiol 354: 3P–22P. 10.1113/jphysiol.1984.sp015397 PMC1193568 6434728

[28] Kelly T, Rose CR (2010) Ammonium influx pathways into astrocytes and neurones of hippocampal slices. J Neurochem 115: 1123–1136. 20854430 10.1111/j.1471-4159.2010.07009.x

[29] Kelly T, Kafitz KW, Roderigo C, Rose CR (2009) Ammonium-evoked alterations in intracellular sodium and pH reduce glial glutamate transport activity. Glia: 921–934. 10.1002/glia.20817 19053055

[30] Stephan J, Haack N, Kafitz KW, Durry S, Koch D, et al (2012) Kir4.1 channels mediate a depolarization of hippocampal astrocytes under hyperammonemic conditions in situ. Glia 60: 965–978. 22431254 10.1002/glia.22328

[31] Rangroo Thrane V, Thrane AS, Wang F, Cotrina ML, Smith NA, et al (2013) Ammonia triggers neuronal disinhibition and seizures by impairing astrocyte potassium buffering. Nat Med 19: 1643–1648. 24240184 10.1038/nm.3400 PMC3899396

[32] Schliess F, Gorg B, Fischer R, Desjardins P, Bidmon HJ, et al (2002) Ammonia induces MK-801-sensitive nitration and phosphorylation of protein tyrosine residues in rat astrocytes. Faseb J 16: 739–741. 11923223 10.1096/fj.01-0862fje

[33] Rose C, Kresse W, Kettenmann H (2005) Acute insult of ammonia leads to calcium-dependent glutamate release from cultured astrocytes, an effect of pH. J Biol Chem 280: 20937–20944. 15802262 10.1074/jbc.M412448200

[34] Jayakumar AR, Rama Rao KV, Tong XY, Norenberg MD (2009) Calcium in the mechanism of ammonia-induced astrocyte swelling. J Neurochem 109 Suppl 1252–257. 19393035 10.1111/j.1471-4159.2009.05842.x PMC4737088

[35] Rose C (2006) Effect of ammonia on astrocytic glutamate uptake/release mechanisms. J Neurochem 97 Suppl 111–15. 16635245 10.1111/j.1471-4159.2006.03796.x

[36] Ohara K, Aoyama M, Fujita M, Sobue K, Asai K (2009) Prolonged exposure to ammonia increases extracellular glutamate in cultured rat astrocytes. Neurosci Lett 462: 109–112. 19576960 10.1016/j.neulet.2009.06.090

[37] Gorg B, Morwinsky A, Keitel V, Qvartskhava N, Schror K, et al (2010) Ammonia triggers exocytotic release of L-glutamate from cultured rat astrocytes. Glia 58: 691–705. 20014275 10.1002/glia.20955

[38] Araque A, Parpura V, Sanzgiri RP, Haydon PG (1999) Tripartite synapses: glia, the unacknowledged partner. Trends Neurosci 22: 208–215. 10322493 10.1016/s0166-2236(98)01349-6

[39] Nedergaard M, Verkhratsky A (2010) Calcium dyshomeostasis and pathological calcium signalling in neurological diseases. Cell Calcium 47: 101–102. 20079921 10.1016/j.ceca.2009.12.011 PMC3268371

[40] Bezzi P, Domercq M, Vesce S, Volterra A (2001) Neuron-astrocyte cross-talk during synaptic transmission: physiological and neuropathological implications. Prog Brain Res 132: 255–265. 11544994 10.1016/S0079-6123(01)32081-2

[41] Nimmerjahn A, Kirchhoff F, Kerr JN, Helmchen F (2004) Sulforhodamine 101 as a specific marker of astroglia in the neocortex in vivo. Nat Methods 1: 31–37. 15782150 10.1038/nmeth706

[42] Kafitz KW, Meier SD, Stephan J, Rose CR (2008) Developmental profile and properties of sulforhodamine 101-labeled glial cells in acute brain slices of rat hippocampus. J Neurosci Methods 169: 84–92. 18187203 10.1016/j.jneumeth.2007.11.022

[43] Kang J, Kang N, Yu Y, Zhang J, Petersen N, et al (2010) Sulforhodamine 101 induces long-term potentiation of intrinsic excitability and synaptic efficacy in hippocampal CA1 pyramidal neurons. Neuroscience 169: 1601–1609. 20600669 10.1016/j.neuroscience.2010.06.020 PMC2918738

[44] Meier SD, Kafitz KW, Rose CR (2008) Developmental profile and mechanisms of GABA-induced calcium signaling in hippocampal astrocytes. Glia 56: 1127–1137. 18442094 10.1002/glia.20684

[45] Helmchen F, Imoto K, Sakmann B (1996) Ca2+ buffering and action potential-evoked Ca2+ signaling in dendrites of pyramidal neurons. Biophys J 70: 1069–1081. 8789126 10.1016/S0006-3495(96)79653-4 PMC1225009

[46] Grynkiewicz G, Poenie M, Tsien RY (1985) A new generation of Ca2+ indicators with greatly improved fluorescence properties. J Biol Chem 260: 3440–3450. 3838314

[47] Rose CR, Karus C (2013) Two sides of the same coin: sodium homeostasis and signaling in astrocytes under physiological and pathophysiological conditions. Glia 61: 1191–1205. 23553639 10.1002/glia.22492

[48] Verkhratsky A KH (1996) Calcium signalling in glial cells. Trends Neurosci 19: 346–352. 8843604 10.1016/0166-2236(96)10048-5

[49] Kirischuk S, Parpura V, Verkhratsky A (2012) Sodium dynamics: another key to astroglial excitability? Trends Neurosci 35: 497–506. 22633141 10.1016/j.tins.2012.04.003

[50] de Knegt RJ, Schalm SW, van der Rijt CC, Fekkes D, Dalm E, et al (1994) Extracellular brain glutamate during acute liver failure and during acute hyperammonemia simulating acute liver failure: an experimental study based on in vivo brain dialysis. J Hepatol 20: 19–26. 7911135 10.1016/s0168-8278(05)80462-3

[51] Michalak A, Rose C, Butterworth J, Butterworth RF (1996) Neuroactive amino acids and glutamate (NMDA) receptors in frontal cortex of rats with experimental acute liver failure. Hepatology 24: 908–913. 8855196 10.1002/hep.510240425

[52] Inesi G, Sagara Y (1994) Specific inhibitors of intracellular Ca2+ transport ATPases. J Membr Biol 141: 1–6. 7966241 10.1007/BF00232868

[53] Golovina VA, Bambrick LL, Yarowsky PJ, Krueger BK, Blaustein MP (1996) Modulation of two functionally distinct Ca2+ stores in astrocytes: role of the plasmalemmal Na/Ca exchanger. Glia 16: 296–305. 8721670 10.1002/(SICI)1098-1136(199604)16:4<296::AID-GLIA2>3.0.CO;2-Z

[54] Beck A, Nieden RZ, Schneider HP, Deitmer JW (2004) Calcium release from intracellular stores in rodent astrocytes and neurons in situ. Cell Calcium 35: 47–58. 14670371 10.1016/s0143-4160(03)00171-4

[55] Verkhratsky A, Orkand RK, Kettenmann H (1998) Glial calcium: Homeostasis and signaling function. Physiol Rev 78: 99–141. 9457170 10.1152/physrev.1998.78.1.99

[56] Rama Rao KV, Jayakumar AR, Norenberg MD (2012) Glutamine in the pathogenesis of acute hepatic encephalopathy. Neurochem Int 61: 575–580. 22285152 10.1016/j.neuint.2012.01.012

[57] Albrecht J, Norenberg MD (2006) Glutamine: a Trojan horse in ammonia neurotoxicity. Hepatology 44: 788–794. 17006913 10.1002/hep.21357

[58] Willard-Mack CL, Koehler RC, Hirata T, Cork LC, Takahashi H, et al (1996) Inhibition of glutamine synthetase reduces ammonia-induced astrocyte swelling in rat. Neuroscience 71: 589–599. 9053810 10.1016/0306-4522(95)00462-9

[59] Dadsetan S, Kukolj E, Bak LK, Sorensen M, Ott P, et al (2013) Brain alanine formation as an ammonia-scavenging pathway during hyperammonemia: effects of glutamine synthetase inhibition in rats and astrocyte-neuron co-cultures. J Cereb Blood Flow Metab 33: 1235–1241. 23673435 10.1038/jcbfm.2013.73 PMC3734774

[60] Chen H, Sun D (2005) The role of Na-K-Cl co-transporter in cerebral ischemia. Neurol Res 27: 280–286. 15845211 10.1179/016164105X25243

[61] Jayakumar AR, Liu M, Moriyama M, Ramakrishnan R, Forbush B 3rd, et al (2008) Na-K-Cl Cotransporter-1 in the mechanism of ammonia-induced astrocyte swelling. J Biol Chem 283: 33874–33882. 18849345 10.1074/jbc.M804016200 PMC2590687

[62] Rodrigo R, Cauli O, Boix J, ElMlili N, Agusti A, et al (2009) Role of NMDA receptors in acute liver failure and ammonia toxicity: therapeutical implications. Neurochem Int 55: 113–118. 19428814 10.1016/j.neuint.2009.01.007

[63] Hermenegildo C, Monfort P, Felipo V (2000) Activation of N-methyl-D-aspartate receptors in rat brain in vivo following acute ammonia intoxication: characterization by in vivo brain microdialysis. Hepatology 31: 709–715. 10706562 10.1002/hep.510310322

[64] Llansola M, Rodrigo R, Monfort P, Montoliu C, Kosenko E, et al (2007) NMDA receptors in hyperammonemia and hepatic encephalopathy. Metab Brain Dis 22: 321–335. 17701332 10.1007/s11011-007-9067-0

[65] Lalo U, Pankratov Y, Parpura V, Verkhratsky A (2011) Ionotropic receptors in neuronal-astroglial signalling: what is the role of “excitable” molecules in non-excitable cells. Biochim Biophys Acta 1813: 992–1002. 20869992 10.1016/j.bbamcr.2010.09.007

[66] Matthias K, Kirchhoff F, Seifert G, Huttmann K, Matyash M, et al (2003) Segregated expression of AMPA-type glutamate receptors and glutamate transporters defines distinct astrocyte populations in the mouse hippocampus. J Neurosci 23: 1750–1758. 12629179 10.1523/JNEUROSCI.23-05-01750.2003 PMC6741945

[67] Latour I, Gee CE, Robitaille R, Lacaille JC (2001) Differential mechanisms of Ca2+ responses in glial cells evoked by exogenous and endogenous glutamate in rat hippocampus. Hippocampus 11: 132–145. 11345120 10.1002/hipo.1031

[68] Attwell D, Buchan AM, Charpak S, Lauritzen M, Macvicar BA, et al (2010) Glial and neuronal control of brain blood flow. Nature 468: 232–243. 21068832 10.1038/nature09613 PMC3206737

[69] Halassa MM, Haydon PG (2010) Integrated brain circuits: astrocytic networks modulate neuronal activity and behavior. Annu Rev Physiol 72: 335–355. 20148679 10.1146/annurev-physiol-021909-135843 PMC3117429

[70] Perea G, Navarrete M, Araque A (2009) Tripartite synapses: astrocytes process and control synaptic information. Trends Neurosci 32: 421–431. 19615761 10.1016/j.tins.2009.05.001

[71] Monfort P, Erceg S, Piedrafita B, Llansola M, Felipo V (2007) Chronic liver failure in rats impairs glutamatergic synaptic transmission and long-term potentiation in hippocampus and learning ability. Eur J Neurosci 25: 2103–2111. 17439494 10.1111/j.1460-9568.2007.05444.x

[72] Chepkova AN, Sergeeva OA, Haas HL (2006) Taurine rescues hippocampal long-term potentiation from ammonia-induced impairment. Neurobiol Dis 23: 512–521. 16766203 10.1016/j.nbd.2006.04.006

[73] Munoz MD, Monfort P, Gaztelu JM, Felipo V (2000) Hyperammonemia impairs NMDA receptor-dependent long-term potentiation in the CA1 of rat hippocampus in vitro. Neurochem Res 25: 437–441. 10823575 10.1023/a:1007547622844

[74] Wen S, Schroeter A, Klocker N (2013) Synaptic plasticity in hepatic encephalopathy - a molecular perspective. Arch Biochem Biophys 536: 183–188. 23624147 10.1016/j.abb.2013.04.008

